# Bloodstream infections in pediatric hematology/oncology patients: a single-center study in Wuhan

**DOI:** 10.3389/fcimb.2024.1480952

**Published:** 2024-12-04

**Authors:** Ibrahim Ahmed Saleh Murshed, Lizhen Zhao, Wenzhi Zhang, Yuhong Yin, Ying Li, Yun Peng, Hongbo Chen, Xiaoyan Wu

**Affiliations:** Department of Pediatrics, Union Hospital, Tongji Medical College, Huazhong University of Science and Technology, Wuhan, China

**Keywords:** bacteremia, hematologic neoplasm, microorganism, neutropenia, pediatric

## Abstract

Bloodstream infections (BSIs) are a critical concern in pediatric onco-hematological patients undergoing chemotherapy or hematopoietic stem cell transplantation (HSCT), leading to a major impact on morbidity, long-term hospitalization, and mortality. We retrospectively analyzed 202 BSIs in 145 patients, consisting of 128 with hematological malignancies, one with a solid tumor, and 16 with non-malignant hematological diseases. We collected data on patient demographics, clinical characteristics, pathogen distribution, and antimicrobial pathogen susceptibility. Gram-positive infections were the most frequent at (58.4%), followed by gram-negative (41%), and fungal infections (0.5%). Particularly, the majority of these infections occurred during the induction phase of chemotherapy, where 94 (46.5%) BSI episodes were recorded, predominantly in neutropenic patients (88.3%). The consolidation phase experienced lower BSIs (11.8%); among these patients (54.1%) were non-neutropenic. BSIs observed in (23.7%) of patients in the maintenance phase, with a higher proportion (66.6%) being neutropenic. Among the 7 patients who underwent HSCT, BSIs occurred in (4.9%) cases, mainly (70%) due to neutropenia. The most prevalent pathogens were Staphylococcus epidermidis (19.8%), Staphylococcus hominis (16.3%), and Escherichia coli (8.4%). The study highlights the critical need for vigilant monitoring and customized infection management strategies to enhance patient outcomes across chemotherapy phases and HSCT.

## 1 Introduction

Advancements in treatment for immunocompromised patients with onco-hematological diseases or stem cell transplantation have reduced mortality and improved survival rates. The significant decrease in adaptive and innate immunity due to underlying diseases and chemotherapy makes these patients highly susceptible to infections with bloodstream infections (BSIs) being a major cause of morbidity and mortality (Islas-Muñoz et al., 2018; Castagnola et al., 2021; Zöllner et al., 2021). Immediate identification and empirical use of antibiotics may enhance the prospects for patients with BSIs. Nevertheless, the selection of empirical antibiotic therapy (EAT) should rely on the patients clinical condition, commonly identified pathogens, and their susceptibility to antimicrobial agents in the specific area (Freifeld et al., 2011).

Treatment of BSIs are a significant issue due to the rising prevalence of antibiotic resistance in multi-resistant bacteria (Russotto et al., 2015). Pediatric patients with cancer across nations are most susceptible to developing multidrug-resistant (MDR) infections. Both gram-positive (GP) and gram-negative (GN) species have shown a global increase in resistance rates (Montassier et al., 2013; Kapoor et al., 2014; Trehan et al., 2014).

To effectively manage BSIs in pediatric onco-hematological patients, it is essential to understand the occurrence of causative microorganisms and their susceptibility to antimicrobials in specific geographical, and temporal contexts. Furthermore, recognize the importance of integrating chemotherapy phase-specific strategies, and hematopoietic stem cell transplantation (HSCT) is crucial for enhancing patient care and improving clinical outcomes. This study aims to investigate the prevalence of specific pathogenic microorganisms and their susceptibility to antimicrobial therapy in a particular region and timeframe among pediatric patients with onco-hematological disorders.

## 2 Methods

### 2.1 Ethical approval

The study was carried out in accordance with the Declaration of Helsinki and approved by the ethics committee of the Union Hospital of Tongji Medical College, Huazhong University of Science and Technology (NO.2024-0529), and patient consent was waived due to the retrospective nature of the cohort study.

### 2.2 Study design and settings

We conducted a retrospective analysis data on children who were admitted to the pediatric hematology and oncology department at Union Hospital, Tongji Medical College, Huazhong University of Science and Technology between January 1, 2020, and December 31, 2023, and had BSIs. Union Hospital’s pediatric hematology and oncology department, a leading healthcare facility in China, focuses on the treatment of pediatric patients with blood disorders and cancer, offering advanced care for children with diverse hematological and oncological conditions.

### 2.3 Case definition

The patients chosen for the medical record review were children aged 0 to 15 years who had positive blood cultures while they were admitted to the hospital. The cases were detected from the microbiology laboratory database. Blood cultures that met the Centers for Disease Control and Prevention (CDC) criteria for contamination were excluded from the study (Horan et al., 2008). Repeat cultures positive for the same bacterium were considered a single infection. True bacteremia cases were defined by the presence of clinical symptoms consistent with infection in addition to a positive blood culture. Patients with positive blood cultures associated with central venous catheters (CVCs), peripherally inserted central catheters (PICCs), or ports were carefully evaluated to distinguish catheter-related infections from contamination.

### 2.4 Data source

We obtained demographic and clinical data from the online patient database and medical records. The recorded outcomes include transfers to the pediatric hematology and oncology departments at Union Hospital. The hematology and oncology department provided data on the total number of inpatient admissions. Data extraction was carried out directly at the hospital.

### 2.5 Study definitions

BSIs were defined as the detection of bacteria or fungus in the blood, confirmed by a positive blood culture, and accompanied by clinical signs and symptoms of infection. Neutropenia is defined as an absolute neutrophil count (ANC) of less than 1.5 x 10^9/L, with severe neutropenia defined as less than 0.5 x 10^9/L. Neutropenia-related infections refer to infections that occur in patients who have neutropenia at the time of the infection.

### 2.6 Statistical analysis

We used descriptive statistical techniques to analyze the demographic and clinical characteristics of pediatric hematology and oncology patients with BSIs. The analysis provided a concise overview of bacterial isolates and their susceptibility to antimicrobial agents. We conducted this analysis and generated outputs using GraphPad Prism and Microsoft Excel. The key factors evaluated included age, gender, phases of chemotherapy, HSCT, levels of minimal residual disease (MRD), types of catheters PICCs, ports, and CVCs, levels of C-reactive protein (CRP), Levels of procalcitonin (PCT), count of white blood cells (WBC), incidence of septic shock, absolute neutrophil count (ANC), and admissions to the intensive care unit (ICU). Correlations between these characteristics and BSI occurrence were investigated.

## 3 Results

### 3.1 Demographics and epidemiology

**Figure 1:**
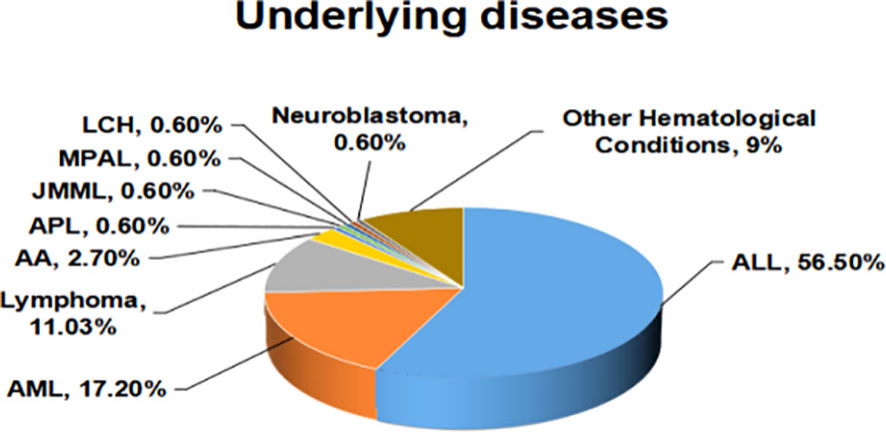
The spread of underlying diseases.

A cohort of 145 onco-hematological pediatric patients, consisting of 90 (62.0%) males and 55 (37.9%) females with a median age of 6.5 years (ranging from 0.11 to 15 years), experienced a total of 202 episodes of BSI. The cohort predominantly consisted of patients with acute lymphoblastic leukemia (ALL), accounting for 82 patients (56.5%). Furthermore, there were 25 patients (17.2%) diagnosed with acute myeloid leukemia (AML), and 16 patients (11.0%) with Lymphoma. **Figure 1** displays the spread of underlying disorders.

GP bacteria were the most predominant, with (32.1%) in ALL patients, (12.8%) in AML patients, (5.9%) in Lymphoma patients, and (7.4%) in other conditions. GN bacteria accounted for (21.7%) in ALL patients, (7.4%) in AML patients, (2.9%) in Lymphoma patients, and (9.4%) in other conditions. Fungi were rare, with only 0.5% in ALL patients. The distribution of microorganisms across diseases are shown in **Table 1**.

**Table 1:** Distribution of microorganisms across diseases.

Diseases								
Microorganisms	ALL		AML		Lymphoma		Others	
	F	%	F	%	F	%	F	%
Gram-positive bacteria	65	32.1	26	12.8	12	5.9	15	7.4
Gram-negative bacteria	44	21.7	15	7.4	6	2.9	18	8.9
Fungi	1	0.5	0	0	0	0	0	0
Total	110	54.3	41	20.2	18	8.8	33	16.3

ALL, Acute Lymphoblastic Leukemia; AML, Acute Myeloid Leukemia; F, Frequency; %, Percentage.

**Figure 2:**
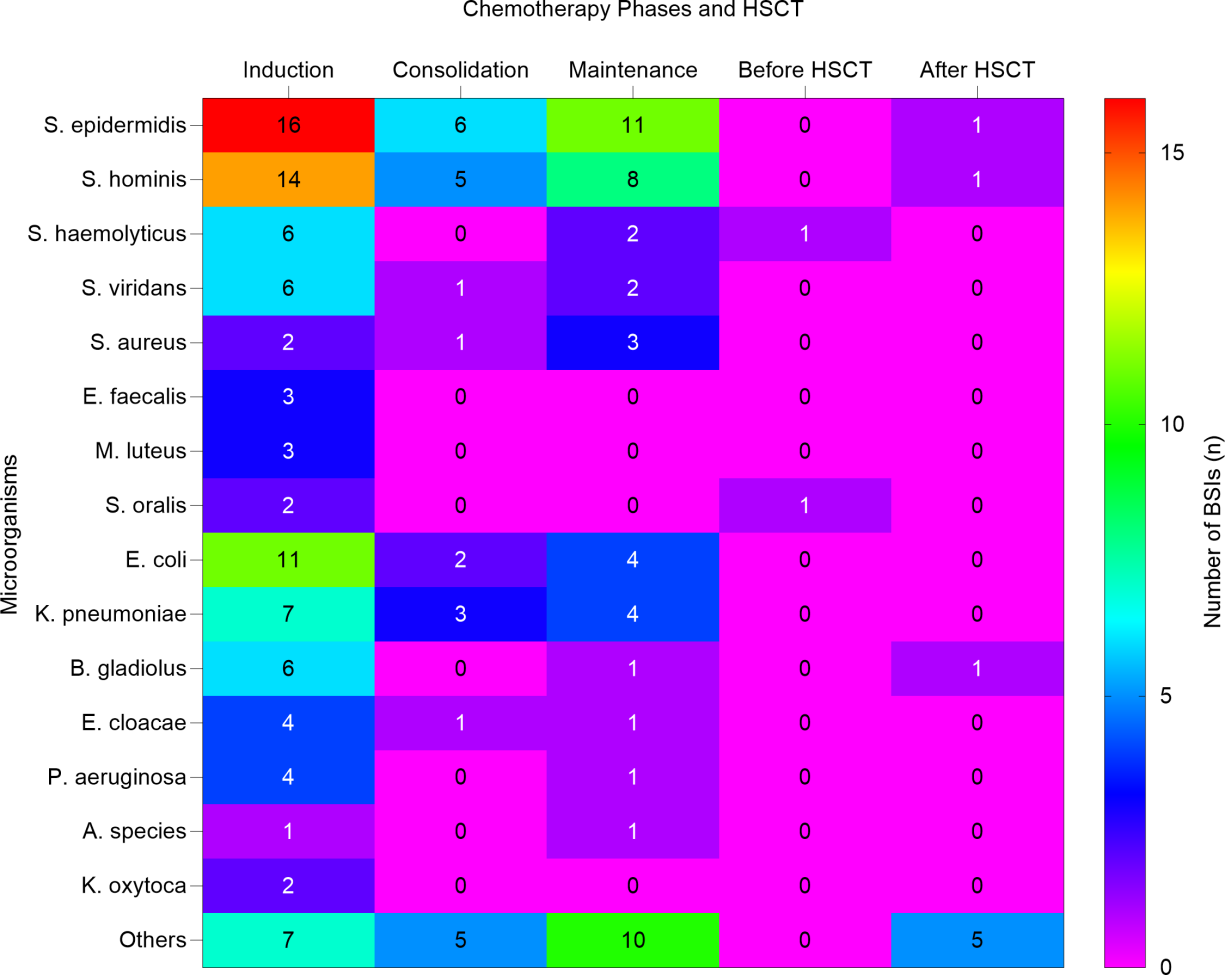
Heat map shows the distribution of bloodstream infection across chemotherapy phases and HSCT. The heat map illustrates the number of bloodstream infections (BSIs) caused by various microorganisms during different phases of chemotherapy and HSCT. *HSCT, Hematopoietic stem cell transplantation. S. epidermidis, Staphylococcus epidermidis; S. hominis, Staphylococcus hominis; S. haemolyticus, Staphylococcus haemolyticus; S. viridans, Streptococcus viridans; S. aureu, Staphylococcus aureus; E. faecalis, Enterococcus faecalis; M. luteus, Micrococcus luteus; S. oralis, Streptococcus oralis; E. coli, Escherichia coli; K. pneumoniae, Klebsiella pneumoniae; B. gladiolus, Burkholderia gladiolus; E. cloacae, Enterobacter cloacae; P. aeruginosa Pseudomonas aeruginosa; A. species, Achromobacter species; K. oxytoca, Klebsiella oxytoca*.

A total of 94 (46.5%) BSI episodes occurred during the induction phase of therapy, predominantly in neutropenic patients (88.3%); 24 (11.8%) during the consolidation phase, among these patients (54.1%) were non-neutropenic; 48 (23.7%) during the maintenance phase, with a higher proportion (66.6%) of these patients being neutropenic; 10 (4.9%) occurred in 7 patients who underwent HSCT, mainly (70%) due to neutropenia. Furthermore, the average hospitalization duration for patients with BSIs ranged from 1 to over 28 days, with the majority of patients (60) staying between 22-28 days. A smaller proportion of patients (20) experienced hospital stays of 1-7 days, while 8-14 days and 15-21 days of hospitalization were observed in 40 and 35 patients, respectively. Prolonged hospitalization (>28 days) occurred in 45 patients. This trend indicates that patients with severe infections, especially those in the induction phase of chemotherapy or those requiring intensive care, tend to experience extended hospital stays. Additionally, 136 (67.3%) of BSIs showed negative MRD, with the majority 73 (36.1%) attributed to the induction phase. Notably, some patients underwent MRD testing multiple times, corresponding to different phases of their treatment. Moreover, 116 (57.4%) of BSIs involved PICC, 58 (28.7%) involved ports, and 2 (1.0%) involved CVC. The induction phase shows the highest proportion of infections, with Staphylococcus epidermidis and Staphylococcus hominis causing sixteen and fourteen BSIs, respectively. During the consolidation phase BSIs decreased with Staphylococcus epidermidis and Staphylococcus hominis identified as the predominant pathogens. In the maintenance phase, Staphylococcus epidermidis was the leading cause of infections. Before HSCT, BSIs were minimal, but after HSCT, a few infections were observed, primarily due to Staphylococcus epidermidis, Burkholderia gladioli, and Staphylococcus hominis. These results highlight the varying prevalence of BSIs during different phases of chemotherapy and HSCT, emphasizing the need for specific infection control strategies during these periods. **Figure 2** describes the distribution of BSIs across chemotherapy phases and HSCT.

The mean and standard deviation (SD) of various infection markers and clinical outcomes were as follows: The mean body temperature of the patients was 38.67 ± 0.81°C, indicating a generally febrile population. Inflammatory markers were also elevated, with the mean CRP level 53.24 ± 58.37 mg/L and PCT 22.20 ± 103.0 µg/L, which shows that the patients had a significant inflammatory response. WBC levels were 2.174 g/L ± 7.229, and ANC levels were 0.8326 x 10^9/L ± 2.066. Notably, there were 17 episodes with an ANC below 1.0 x 10^9/L and 139 episodes below 0.5 x 10^9/L, highlighting the prevalence of severe neutropenia in these patient populations. The presence of severe neutropenia is significant, which increases the risk of infection and complicates BSI management. During the BSI episodes, 9 patients (6.2%) developed septic shock, and 15 patients (10.3%) required ICU admission, indicating the severity of these infections and the necessity for intensive medical care. The summarized demographic and clinical features of patients are presented in **Table 2**.

**Table 2:** Demographic and clinical features of patients with bloodstream infections.

Characteristic	Total n
Total patients	145
Age (median, range), years	6.5 (0.11-15)
Male n (%)	90 (62.0)
Body temperature (°C) at the beginning of BSI Mean (SD)	38.67 ± 0.81
C-Reactive Protein (CRP, mg/L)	53.24 ± 58.37
Procalcitonin (PCT, µg/L)	22.20 ± 103.0
White Blood Cells (WBC, g/L)	2.174 ± 7.229
Absolute Neutrophil Count (ANC)	0.8326 x 10^9/L ± 2.066
Episodes with ANC < 1.0 x 10^9/L	17
Episodes with ANC < 0.5 x 10^9/L	139
Catheter related bloodstream infections	176
Catheter type	–
BSIs involving PICC	116 (57.4%)
BSIs involving Ports	58 (28.7%)
BSIs involving CVC	2 (1.0%)
Patients with Septic Shock	9 (6.2%)
ICU Admissions	15 (10.3%)

### 3.2 Blood culture results

During the study period, we recorded 202 BSIs in 145 patients. GP BSIs made up 58.4% (118/202) of all episodes; GN BSIs made up 41% (83/202); and fungal organisms made up 0.5% (1/202) of all positive blood culture isolates. Of the 202 BSIs, (57.4%) were associated with PICCs, (28.7%) with ports, and (1%) with CVCs. All cases were confirmed as true bacteremia based on clinical symptoms consistent with infection and microbiological findings. Blood cultures that met CDC criteria for contamination were excluded, ensuring that only clinically significant infections were included in the final analysis. The predominant GP isolates were Staphylococcus epidermidis (33.9%), and Staphylococcus hominis (16.3%), while Escherichia coli (20.4%) and Klebsiella pneumoniae (18%) dominated among GN pathogens. **Table 3** summarizes the most common causative agents for bloodstream infections.

**Table 3:** The most common causative agents for bloodstream infections.

Gram-Positive	Episodes (*N* = 118), No. (%)
Staphylcoccus epidermidis	40 (19.8)
Staphylococcus hominis	33 (16.3)
Staphylococcus haemolyticus	11 (5.4)
Streptococcus viridans	9 (4.4)
Staphylococcus aureus	6 (2.9)
Enterococcus faecalis	3 (1.4)
Micrococcus luteus	3 (1.4)
Sterptococcus oralis	2 (0.9)
Others	11 (5.4)
Gram-Negative	Episodes (*N* = 83), No. (%)
Escherichia coli	17 (8.4)
Klebsiella pneumoniae	15 (7.4)
Burkholderia gladiolus	15 (7.4)
Enterobacter cloacae	9 (4.4)
Pseudomonas aeruginosa	7 (3.4)
Achromobacter species	3 (1.4)
Klebsiella oxytoca	3 (1.4)
Serratia marcescens	3 (1.4)
Acinetobacter baumannii	2 (0.9)
Stenotrophomonas maltophilia	2 (0.9)
Others	7 (3.4)
Fungi	Episodes (*N* = 1), No. (%)
Candida albicans	1 (0.5)

### 3.3 Antimicrobial susceptibility

**Figure 3:**
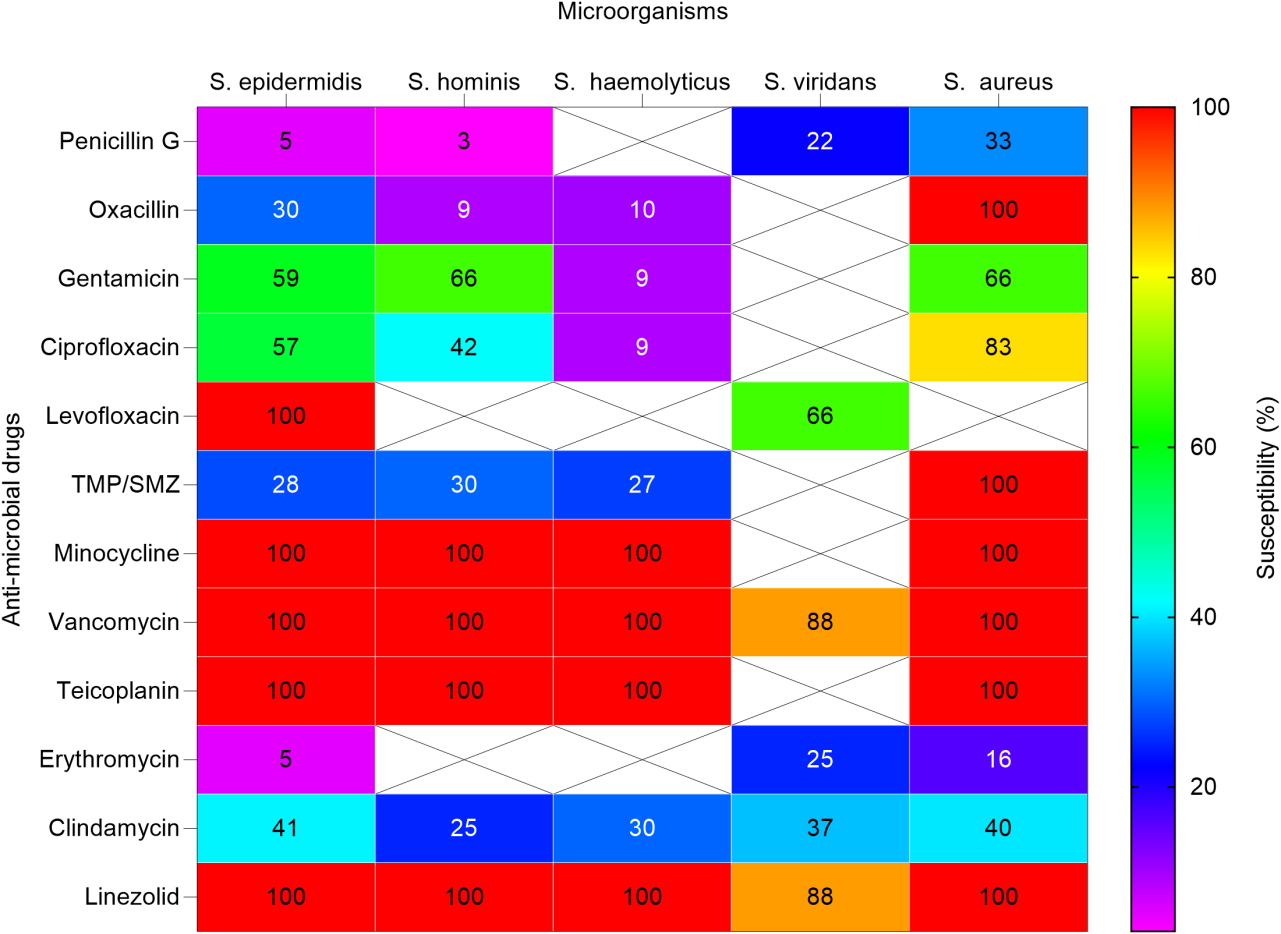
Heat map shows the susceptibility patterns of gram-positive isolates from infected children (2020-2023). Susceptibility patterns of pathogens isolated more than once are reported. The color scale on the right represents the percentage of susceptibility. Crossed cells (X) indicate that the specific anti-microbial drug was not tested against the corresponding microorganism. *S. epidermidis, Staphylococcus epidermidis; S. hominis; Staphylococcus hominis; S. haemolyticus, Streptococcus haemolyticus; S. viridans; Streptococcus viridans; S. aureus, Staphylococcus aureus*. In some cases, isolates were not tested against antimicrobial agents normally used.

**Figure 4:**
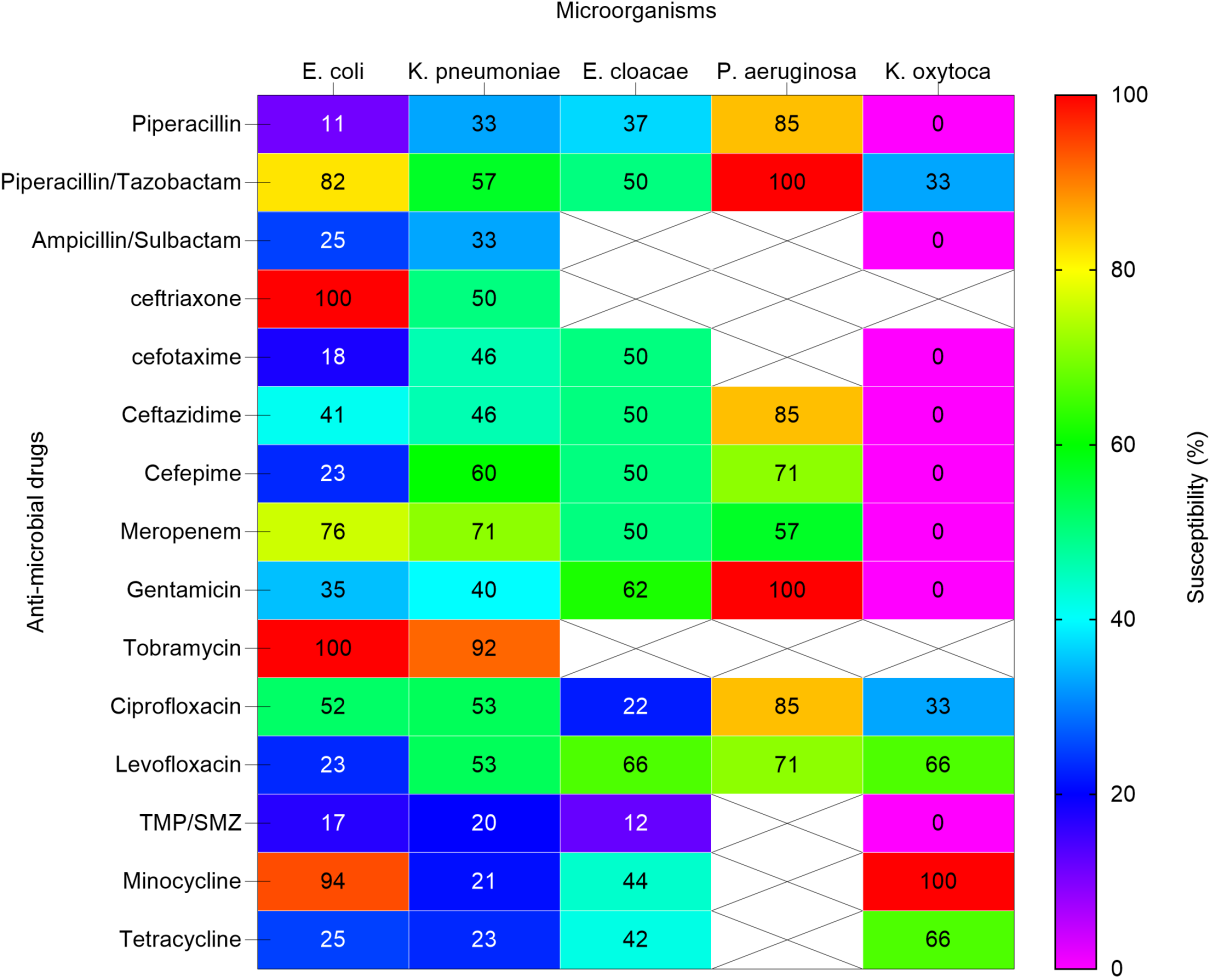
Heat map shows the susceptibility patterns of gram-negative isolates from infected children (2020-2023). Susceptibility patterns of pathogens isolated more than once are reported. The color scale on the right represents the percentage of susceptibility. Crossed cells (X) indicate that the specific anti-microbial drug was not tested against the corresponding microorganism. *E. coli, Escherichia coli; K. pneumoniae subspecies pneumoniae, Klebsiella pneumoniae subspecies pneumoniae; E. cloacae, Enterobacter cloacae. P. aeruginosa, Pseudomonas aeruginosa; K. oxytoca, Klebsiella oxytoca; TMP/SMZ, Trimethoprim / Sulfamethoxazole*.

Anti-microbial susceptibility profiles for various bacterial isolates are presented in **Figures 3**, **4**. Over (94%) of Staphylococcus epidermidis isolates were not sensitive to penicillin G. However, all GP isolates were sensitive to levofloxacin, minocycline, vancomycin, teicoplanin, and linezolid. Staphylococcus hominis were very sensitive to minocycline, vancomycin, and linezolid, but (97%) were resistant to penicillin G and (91%) to oxacillin. Staphylococcus haemolyticus was susceptible to several antimicrobial agents, although gentamicin and trimethoprim/sulfamethoxazole (TMP/SMZ) showed mild effectiveness.

Escherichia coli demonstrated significant MDR, with low susceptibility to penicillins such as piperacillin (11%) and cephalosporins including ceftriaxone (0%) and ceftazidime (41%). However, higher susceptibility was observed to piperacillin/tazobactam (82%) and tobramycin (100%).

Klebsiella pneumoniae showed resistance to ampicillin/sulbactam (33%) and piperacillin (33%), but retained susceptibility to tobramycin (92%) and meropenem (71%). These results highlight the MDR profiles of these pathogens, which pose challenges in selecting effective empirical therapies.

The streptococcus viridans showed high susceptibility to vancomycin and linezolid, as well as mild susceptibility to levofloxacin. Staphylococcus aureus showed high susceptibility to oxacillin, minocycline, vancomycin, and linezolid but variable susceptibility to other antibiotics.

In our study, Escherichia coli isolates were resistant to piperacillin, but showed high susceptibility to piperacillin-tazobactam and tobramycin. Klebsiella pneumoniae showed the highest susceptibility to tobramycin and meropenem. Susceptibility data for burkholderia gladiolus was not available. Enterobacter cloacae showed moderate to high susceptibility to meropenem, gentamicin, and levofloxacin. Pseudomonas aeruginosa showed significant susceptibility to piperacillin/tazobactam, gentamicin, and tobramycin.

Penicillin G and oxacillin are becoming less effective and may no longer be reliable treatments for these GP infections due to observed high resistance rates. Linezolid showed efficacy against many staphylococcal infections, including staphylococcus aureus and staphylococcus hominis, indicating its potential as a key drug for treating resistant staphylococcal infections. However, continuous monitoring for emerging resistance is crucial.

### 3.4 Empirical antibiotic treatment and resistance

The findings highlight rates of resistance among GP isolates, particularly against penicillin G and oxacillin. This suggests that these antibiotics efficacy in infection treatment is declining. However, the susceptibility of these isolates to alternative agents like levofloxacin, minocycline, vancomycin, teicoplanin, and linezolid emphasizes the need to explore alternative treatment avenues. Continuous monitoring for emerging resistance is crucial for maintaining effective infection management strategies.

## 4 Discussion

Bloodstream infections are a significant concern in pediatric patients undergoing chemotherapy or HSCT, often leading to prolonged hospitalization, increased morbidity, and mortality (Islas-Muñoz et al., 2018).

The prevention of BSIs is critical, particularly in vulnerable populations such as pediatric patients undergoing chemotherapy or HSCT. Prophylaxis strategies play a significant role in reducing the incidence of BSIs. Current clinical guidelines recommend the use of antimicrobial prophylaxis, including fluoroquinolones, to mitigate the risk of bacterial infections in neutropenic patients (Lehrnbecher et al., 2020). However, the widespread use of prophylactic antibiotics has also contributed to the emergence of antibiotic resistance, particularly in Gram-negative and Gram-positive bacteria (Karaman et al., 2020; Kakoullis et al., 2021).

Early recognition of infection is essential in reducing morbidity and mortality. Neutropenic patients should be closely monitored for early signs of infection, such as febrile episodes, changes in clinical status, or abnormal laboratory results like elevated CRP and PCT levels. These indicators can help identify infections before they progress to severe sepsis or septic shock, allowing for the timely initiation of empirical antibiotic therapy (Massaro et al., 2007; Cruz et al., 2020).

This work investigates the epidemiology of BSIs among pediatric onco-hematological patients at a single center in Wuhan over four years, focusing on the prevalence of specific pathogens, their susceptibility to antimicrobial therapy, and the impact of different phases of chemotherapy and HSCT on infection rates. Our findings revealed that GP organisms were the predominant causative agents of BSIs, accounting for (58.4%) of all episodes, followed by GN bacteria at (41%), and fungal BSIs were rare, constituting of (0.5%) of cases. This distribution aligns with previous studies highlighting the dominance of GP bacteria in pediatric onco-hematological related infections (Dutta and Flores, 2018; Zhu et al., 2018; Youssef et al., 2020; Castagnola et al., 2021; Zöllner et al., 2021; Mattei et al., 2022).

In the 1960s and 1970s, BSIs were mostly caused by GN bacteria. However, since the mid-1980s, there has been a shift with GP bacteria becoming more common (Gustinetti and Mikulska, 2016). The use of single-agent antibiotic therapy for febrile neutropenia is risky as it increases the chance of treatment failure due to antibiotic resistance (Mikulska et al., 2014; Lehrnbecher et al., 2017). Since 2000, bacteria with Extended-Spectrum Beta-Lactamase (ESBL) genes have become much more common. These genes can deactivate powerful antibiotics like third and fourth-generation cephalosporins and antipseudomonal penicillins. This has led to increased reliance on carbapenems in clinical settings (Nordmann et al., 2011; Darby et al., 2023). This change has resulted in an increased occurrence of bacterial strains that have the ability to produce carbapenemases, which are enzymes that can degrade carbapenems (Tzouvelekis et al., 2012; Elshamy and Aboshanab, 2020).

In comparison with the findings from other research (Knight et al., 2019), our study showed that all GN organisms were intermediate susceptible to carbapenems. These differences between various studies highlight the critical need for localized surveillance as antibiotic resistance patterns can significantly differ across regions and institutions (Knight et al., 2019).

By 2000, the prevalence of coagulase-negative staphylococci (CoNS) as a cause of BSIs in the United States had increased to (76%) due to the widespread administration of fluoroquinolone antibiotics for prophylaxis. The most frequently identified strains were CoNS, Viridans streptococci, and enterococci (Wisplinghoff et al., 2003).

In recent years, various variables have played a role in the growth of GN isolates. These include improved management of CVC, the appearance of fluoroquinolone-resistant GN bacteria, and alterations in chemotherapy protocols resulting in higher levels of gastrointestinal toxicity and endogenous microbial infections (Collin et al., 2001; Gudiol et al., 2013; Garcia-Vidal et al., 2018).

In this study, GP bacteria isolates were the most prevalent, accounting for (58.4%) of cases, with Staphylococcus epidermis being the most common. In contrast, GN bacteria accounted for (41%) of isolates, with Escherichia coli and Klebsiella pneumoniae being the most frequent. These observed patterns are consistent with conclusions findings from similar studies, suggesting a potential correlation with reduced selective burden on GN intestinal flora due to the absence of fluoroquinolone prophylaxis. Additionally, the widespread use of CVC in pediatric populations may also contribute to these findings (Ramphal, 2004; Lakshmaiah et al., 2015; Trecarichi et al., 2015; Gustinetti and Mikulska, 2016).

The induction phase of chemotherapy emerged as the period with the highest prevalence of BSIs, affecting (46.5%) of patients with onco-hematological diseases. Neutropenia-related infections were common during this phase, emphasizing the vulnerability of patients with compromised immune systems to bacterial invasion. Furthermore, this data highlight the importance of vigilant monitoring and infection prevention strategies during the induction phase to reduce the risk of BSIs and associated complications. In terms of specific pathogens, Staphylococcus epidermidis was the most frequently isolated GP organism, consistent with its function as a common commensal organism and a significant opportunistic pathogen in immunocompromised individuals (Freifeld et al., 2011). In our study, we took particular care to differentiate between contamination and true bacteremia, especially with pathogens like Staphylococcus epidermidis, commonly associated with catheter-related infections. By carefully evaluating the type of catheter used (PICCs, ports, CVCs) and confirming clinical symptoms consistent with infection, we ensured that only clinically significant bacteremias were included in the final analysis, and any potential contamination was excluded. This approach minimized the risk of misclassification and provided a more accurate reflection of infection rates in our pediatric hematology/oncology patients cohort. Among GN bacteria, Escherichia coli and Klebsiella pneumoniae were the predominant species, reflecting the global trend of increasing resistance among Enterobacteriaceae in healthcare facilities (Russotto et al., 2015).

Analysis of antimicrobial susceptibility patterns revealed concerning rates of resistance among GP isolates, particularly against penicillin G and oxacillin. This highlights the urgent need for appropriate antibiotic use and continuous surveillance of resistance patterns to guide empirical therapy effectively.

Additionally, our study identified significant MDR among GN organisms, particularly Escherichia coli and Klebsiella pneumoniae, with resistance to multiple classes of antibiotics, including penicillins and cephalosporins. The rising prevalence of MDR strains complicates the treatment of BSIs, particularly in pediatric onco-hematological patients. The MDR patterns emphasize the need for customized empirical therapies based on local resistance patterns, along with the implementation of antimicrobial stewardship programs to mitigate the development of further resistance.

Effective management of BSIs in pediatric onco-hematological patients requires a multi-pronged approach, combining prophylaxis, timely diagnosis, and targeted therapy. Prophylactic strategies, such as the administration of fluoroquinolones during periods of profound neutropenia, have been shown to reduce the incidence of bacterial infections (Gafter-Gvili et al., 2012). However, the increased use of antimicrobial prophylaxis has also led to antibiotic resistance, as demonstrated by the high resistance rates in Staphylococcus epidermidis and Escherichia coli in our cohort.

The rising incidence of MDR organisms, such as Escherichia coli and Klebsiella pneumoniae, poses significant challenges in the empirical management of BSIs. Current empirical therapy often involves broad-spectrum antibiotics, but the emergence of MDR strains necessitates the prudent use of antibiotics and antimicrobial stewardship programs to prevent further resistance (Kakoullis et al., 2021).

In our study, linezolid, vancomycin, and levofloxacin were effective against GP organisms such as Staphylococcus hominis and Staphylococcus epidermidis, whereas piperacillin-tazobactam and meropenem showed efficacy against GN pathogens. The importance of revising empirical regimens based on local susceptibility patterns cannot be understated, especially given the high prevalence of MDR bacteria. Once a pathogen is identified through blood cultures or rapid diagnostic methods, transitioning from broad-spectrum empiric therapy to pathogen-targeted therapy is critical to improving outcomes while minimizing the development of further resistance (Magiorakos et al., 2012; Tamma et al., 2012).

Antimicrobial stewardship programs are essential in this context, as they guide the appropriate use of antibiotics and help reduce the selective pressures that drive resistance. Implementing standardized protocols for infection control, including hand hygiene, catheter care, and early removal of unnecessary invasive devices, can also significantly reduce the incidence of catheter-related BSIs (Karanika et al., 2016).

This study emphasizes the importance of alternative agents such as levofloxacin, minocycline, vancomycin, teicoplanin, and linezolid, which showed higher efficacy against resistant GP pathogens. In contrast, GN bacteria showed variable susceptibility profiles, with some strains showing resistance to multiple antimicrobial classes. Notably, pseudomonas aeruginosa showed high susceptibility to piperacillin/tazobactam, gentamicin, and tobramycin, suggesting the continued effectiveness of these agents in treating Pseudomonas infections. However, the emergence of resistance highlights the importance of ongoing surveillance and antimicrobial stewardship efforts.

The EAT strategy discussed here predominantly consists of broad-spectrum regimens, reflecting the current standard of care for febrile neutropenia in pediatric oncology patients (Trehan et al., 2014). However, the rising prevalence of MDR organisms poses a significant challenge to empirical therapy, requiring an advanced approach to antibiotic selection based on local epidemiology and antimicrobial susceptibility data (Ramphal, 2004; Gudiol et al., 2013; Montassier et al., 2013; Trecarichi et al., 2015; Al-Tawfiq et al., 2019; Martinez-Nadal et al., 2020).

A detailed understanding of local infection epidemiology is vital for optimizing the selection of antibiotic therapy in the management of febrile neutropenia (Martinez-Nadal et al., 2020). The selection of antibiotics varies according to the febrile neutropenia management policy implemented. The escalation policy, which involves initiating treatment with monotherapy targeting primarily GN bacteria and subsequently adding additional antibiotics if the patient’s condition worsens, presents several limitations. These limitations include inadequate coverage for both GN and GP bacteria, such as CoNS and streptococci, as well as an increased risk of treatment failure due to bacterial antibiotic resistance (Holland et al., 2014; Blennow and Ljungman, 2016). Conversely, the de-escalation policy involves starting with broad-spectrum antibiotics and then adjusting the therapy based on microbiology results until the patient’s neutrophil count recovers and/or there is a significant clinical improvement. While these approaches are firmly established for adult cancer patients, especially those dealing with severe sepsis in the ICU (Mokart et al., 2014; Routsi et al., 2020), Data regarding the escalation approach in pediatric patients is limited (Meryk et al., 2021).

This study has several strengths, including a comprehensive analysis of BSIs over four years and a detailed characterization of pathogenic microorganisms and their susceptibility profiles. However, certain limitations must be acknowledged, including the retrospective nature of the study, potential biases inherent in data collection, and the single-center design, which may limit the generalizability of our findings. Additionally, the relatively small sample size and the four-year study duration may have constrained the statistical power and comprehensiveness of our conclusions. Expanding the sample size and conducting multi-center research could further enhance the reliability and applicability of the findings to a broader population. Further research is warranted to explore the efficacy of targeted antimicrobial therapies and the role of antimicrobial management programs in optimizing outcomes for pediatric patients with hematological malignancies undergoing chemotherapy or HSCT.

In conclusion, our study provides valuable insights into the epidemiology of BSIs among pediatric onco-hematological patients at a single center in Wuhan. The predominance of GP organisms, high rates of resistance among GP isolates, and the impact of different phases of chemotherapy on infection rates highlighting the importance of customized infection prevention and management strategies in this vulnerable patient population.

## Data Availability

The raw data supporting the conclusions of this article will be made available by the authors, without undue reservation.
